# Amlodipine and Baclofen Overdose Causing Cardiogenic Shock and Mimicking Brain Death: A Novel Combination

**DOI:** 10.7759/cureus.96470

**Published:** 2025-11-10

**Authors:** Farhan Z Alenezi, Bader M Alasmari, Yasser A Alrumih, Ahmad A Alrukban, Saad Alqahtani

**Affiliations:** 1 Department of Intensive Care Medicine, King Abdulaziz Medical City, Riyadh, SAU; 2 College of Applied Medical Sciences, King Saud Bin Abdulaziz University for Health Sciences College of Medicine, Riyadh, SAU

**Keywords:** baclofen toxicity, brain death mimicry, calcium channel blocker toxicity, medication side effects, neurology and critical care, suicidal attempt

## Abstract

Suicide has always been a global health crisis that affects every population on earth. For as long as it has been recorded, overdosing and taking herbal medicines have been a common way to commit suicide. Advancements in pharmaceuticals and the readily available nature of over-the-counter (OTC) medications have led to the prevalence of using OTC medications as a means to end one’s life. Of these medications, cardiac medications, i.e., heart failure and hypertension medications, constitute one of the most potent forms taken for committing suicide. We present a case of a combined toxicity where a gentleman attempted suicide by taking a combination of amlodipine and baclofen, which caused severe cardiogenic shock followed by apparent clinical brain death, preventing the use of extracorporeal support, later resolving with complete resolution without any residual deficit, with the patient confirming the ingestion of baclofen upon waking up. This is the first encounter with such a combination to be recorded in the literature with complete resolution of symptoms.

## Introduction

Suicide, the act of taking one’s life, is considered a global health crisis as a leading preventable cause of death. Suicidal attempts represent a major burden on healthcare systems worldwide [1]. Suicide is the 18th leading cause of death according to the World Health Organization [2]. Suicide is still considered a major social taboo within Saudi Arabia, which follows Sharia law, in which suicide is viewed with substantial prejudice, both socially and religiously [3]. While these factors remain at play, suicide is not completely absent within Saudi Arabia. With the complexity of changing demographics, influx of international visitors and expatriate workers, and increase in psychiatric awareness, the topic of self-inflicted harm is becoming more openly discussed around the Kingdom. Recent evidence suggests that the rate of suicide and self-inflicted harm has been slowly increasing over the past 30 years [4]. Many methods to commit suicide have been documented, but according to the Centers for Disease Control and Prevention, poisoning remains among the top three methods [5]. Antihypertensive medications and heart failure medications, specifically calcium channel blockers and beta-blockers, represent some of the most common offenders. Combination toxicities, i.e., ingesting multiple medications, are more fatal due to the complicated pharmacological interactions and difficulty in detecting some of these combinations unless clearly stated by the patient. These combinations can be quite variable and often reflect the serious intention of the drug taker to commit suicide, as some of these medications exacerbate the effects of their co-ingested counterparts. Of these combinations, the most serious risk stems from combinations that cause cardiovascular and neurological depressing effects, as they can be attributed to each other’s effects, can pose serious life-threatening risk, and can be challenging to manage. Here, we present a novel case of a patient who presented to the hospital following a calcium channel blocker overdose with suicidal intent that resulted in severe refractory vasoplegic shock, with additional baclofen toxicity mimicking brain death.

## Case presentation

A 45-year-old male with a known history of hypertension on amlodipine and valsartan, dyslipidemia on atorvastatin, and a previous basal ganglia hemorrhagic stroke around one year ago presented to the emergency department (ED) with a low level of consciousness. The patient was of Nepalese origin and could not speak English or Arabic. He was found by his roommates in a deranged state from his usual self, with deep snoring and thick secretions lurking within the oral cavity. Upon arrival at the hospital, the roommate confirmed he was last seen in a normal state around 1400, and that four hours had elapsed since then. Hence, a stroke code was called due to presentation and previous history, and the patient was intubated immediately.

**Figure 1:**
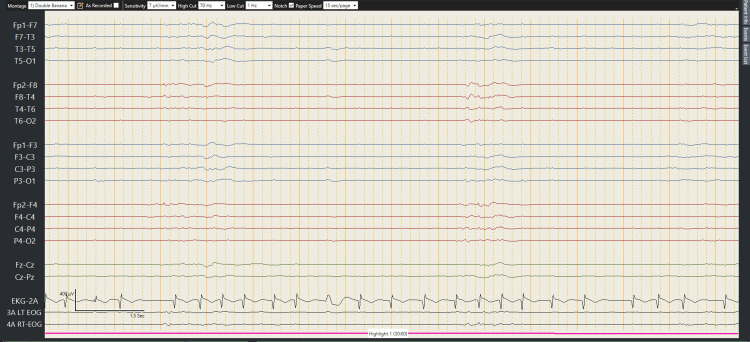
Initial electroencephalogram showing severe diffuse encephalopathy with nearly no electrical brain activity, taken while the patient was off sedation completely within the first 24 hours.

The initial examination revealed an obtunded middle-aged male with average body build, un-arousable and unresponsive, sluggishly responsive 2 mm pupils bilaterally, with no obvious focal deficit or identifiable abnormality on neurological examination. He was noted to be hypertensive initially with fluctuating heart rate and a random blood glucose of 8.7 mmol/L. He was rushed to the Radiology Department, where he underwent multiple CT studies, including brain CT, brain angiography CT, and a brain perfusion CT, which yielded no clear explanation for his altered level of consciousness. There were findings suggestive of a prior insult that were attributed to his previous hemorrhagic stroke one year ago. He was loaded with antiepileptic medications, levetiracetam 60 mg/kg, and a lumbar puncture was performed, along with an urgent electroencephalogram (EEG). At this point, he was not a candidate to receive tissue plasminogen activator or thrombectomy. An acute infectious process was suspected as well as an epileptic crisis. While a toxicologic emergency was suspected, the overall presentation at that point was not clearly consistent with a toxidrome. Nonetheless, a toxicologic workup was requested, and thiamine was administered. The intensive care unit (ICU) team was involved early in the case due to the patient’s state, and he was planned for admission to the neurocritical care unit. As the EEG showed severe diffuse encephalopathy with high risk for seizures, an additional load of valproic acid was administered. At this point, the patient was noted to have fixed dilated pupils of 5 mm, with hypotension mandating the use of vasoactive medications. He was kept on norepinephrine, and vasopressin was added to achieve a mean arterial pressure (MAP) of 65 mmHg or higher. Sedation was kept off due to the new changes in his level of consciousness, and an arterial line was inserted to measure blood pressure more accurately, which confirmed severe hypotension despite vasoactive medications. The EEG (Figure 1) confirmed a severe diffuse encephalopathy.

The patient’s blood gas and metabolic panels (Table 1) kept worsening. His shock state mandated the full support of four vasoactive medications (norepinephrine, epinephrine, vasopressin, and phenylephrine) to maintain a MAP of 65mmHg or greater, with lactic acidosis reaching a level of 22 mmol/L. He developed an acute kidney injury with severe metabolic acidosis, pH of 7.01, and bicarbonate of 8 mmol/L, as well as a severe, uncontrollable hyperglycemia and hyperosmolar state. Cerebrospinal fluid (CSF) analysis and a biochemical profile had been completed at this time.

**Table 1:** Laboratory findings after the initial resuscitation. All items preceded by cerebrospinal fluid (CSF) were obtained from CSF. Viral multiplex and cultures were negative.

Parameter	Result	Reference range
Blood gas pH	7.01	7.35–7.45
Blood gas bicarbonate (HCO_3_, mmol/L)	8.3	22–26
Blood gas (pCO_2_, mmHg)	33	35–45
Blood gas (pO_2_, mmHg)	103	75–100
White blood cell count (10^9^/L)	17.0	4.5–10.0
Hemoglobin (g/dL)	13.7	13.0–17.0
Platelet count (10^9^/L)	144	150–400
Glucose (mmol/L)	33.8	3.9–5.8
Creatinine (mmol/L)	174	60–110
Sodium (mmol/L)	149	135–145
Potassium (mmol/L)	2.3	3.5–5.1
Chloride (mmol/L)	103	98–107
Blood urea nitrogen (mmol/L)	5.9	2.5–7.1
Lactic acid (mmol/L)	22.38	0.5–2.2
CSF appearance	Clear	Clear, colorless
CSF white blood cells (cells/µL)	1	0–5
CSF red blood cells (cells/µL)	1,578	0
CSF glucose (mmol/L)	5.3	2.2–3.9
CSF protein (g/L)	0.61	0.15–0.45
CSF lactic acid (mmol/L)	3.19	1.1–2.4

The patient was started on an insulin infusion for profound hyperglycemia and suspected calcium channel blocker toxicity, and electrolytes were monitored every four hours with multiple derangements requiring frequent electrolyte corrections. At this point, the patient was off sedation for around 18 hours with no response, in severe shock on four vasoactive medications with no clear etiology, and deeply comatose. The pupils were sluggishly reactive at 2 mm bilaterally, with absent reflexes and evident cough, gag, and corneal reflexes. CSF bacterial cultures and viral multiplex were negative. EEG was repeated and was consistent with a high epileptiform burst, suggestive of significant cortical excitability and a diffuse profound encephalopathy with no definite focality or origin. The treating team had maximized all vasoactive medications and kept the patient on two antiepileptic medications, and added a third line, lacosamide, as well as a plan for long-term EEG monitoring for 24 hours.

The patient’s clinical condition denied access to any further imaging. Extracorporeal membrane oxygenation (ECMO) was considered as a last resort and the ECMO team were contacted for possible cannulation and veno-arterial ECMO support; however, he was not a candidate due to neurological condition and expected poor prognosis due to poor neurological function at that time, evident by fixed dilated pupils, no gag reflex, no cough reflex, and previously mentioned EEG tracing (Figure 1). The attending toxicologist was contacted initially and advised to treat for a possible cardio-active medication toxicity, although with low likelihood, but to start treatment if he was persistently hypotensive. A review of home medications showed that the patient was on amlodipine and valsartan and was taking baclofen for muscular pain of unclear origin; hence, lipid emulsion therapy was given twice with partial response. He was also treated with methylene blue, receiving a total dose of 100 mg. The patient’s requirements continued to be extremely high in terms of hemodynamic support, and he continued to experience severe electrolyte derangements as well as refractory hyperglycemia. The insulin infusion dose was escalated accordingly, with some response. After ruling out other major possible causes of vasoplegic shock, the patient was started on treatment for calcium channel blocker toxicity. He was placed on high-dose insulin infusion, reaching a maximum of 55 U/hour. The patient’s neurological condition continued to be the same for the next two days, with mild improvements by the end of day three of admission in the form of agitation and tachycardia, with long-term EEG monitoring consistently showing diffuse profound encephalopathy with no epileptic discharges. The hemodynamic support was weaned after the introduction of insulin, and lactic acidosis started to improve, going down to 2.2 mmol/L, and so did the kidney function, normalizing from a creatinine level of 174 mmol/L to a range of 60 mmol/L over the course of seven days. The biochemical profile began to show signs of improvement (Table 2) as the patient was on high-dose insulin infusion.

**Table 2:** Laboratory findings on day three of intensive care unit admission after better stabilization while on insulin infusion at 55 U/hour.

Parameter	Result	Reference range
Blood gas pH	7.40	7.35–7.45
Blood gas bicarbonate (HCO_3_, mmol/L)	20.4	22–26
Blood gas (pCO_2_, mmHg)	33	35–45
Blood gas (pO_2_, mmHg)	71	75–100
White blood cell count (10^9^/L)	11.2	4.0–10.0
Hemoglobin (g/dL)	12.1	13.0–17.0
Platelet count (10^9^/L)	144	150–400
Glucose (mmol/L)	36.3	3.9–5.8
Creatinine (mmol/L)	102	60–110
Sodium (mmol/L)	146	135–145
Potassium (mmol/L)	4.1	3.5–5.1
Chloride (mmol/L)	112	98–107
Blood urea nitrogen (mmol/L)	4.1	2.5–7.1
Lactic acid (mmol/L)	9.8	0.5–2.2

**Figure 2:**
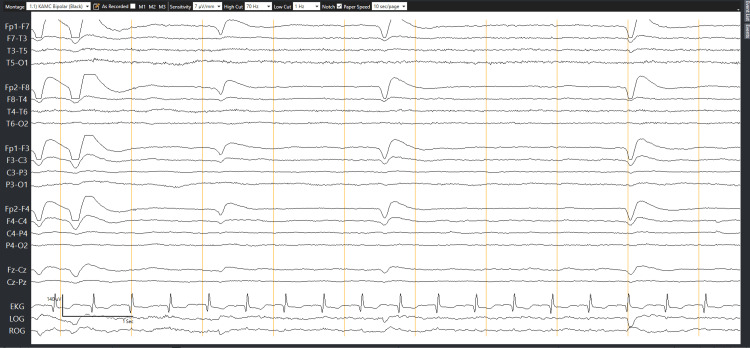
Repeat electroencephalogram following improvement in the level of consciousness and meaningful response from the patient.

The EEG was repeated on the third day (Figure 2) when the patient began to regain consciousness, and showed similar findings as the previous EEG, albeit with minimal improvement. He required six doses of intravenous (IV) calcium during the first two days, and no infusion was started.

A repeat CT of the brain was done on day seven of admission, with no acute worsening of the initial injury or changes from baseline CT apart from a mild opacification of the sinuses. A brain MRI was performed on day nine, with no acute insults to explain the neurological condition of the patient, only showing mucosal thickening of the sinuses bilaterally and the previously known basal ganglia injuries from the prior hemorrhagic stroke. Following MRI, the sedation was weaned down to prepare for extubation as the neurological condition had improved, and the patient was extubated two days later with full regain of neurological function on day 11 of admission. Following extubation, the patient was questioned as to what he had ingested, and confirmed that he had ingested around 40 tablets of all medications that were available to him, which included amlodipine and baclofen, with suicidal intent due to a severe psychotic disorder of unclear origin. Further characterization of his psychiatric condition was restricted at that time due to a language barrier and ICU-acquired delirium, and he was discharged to the floor with close monitoring on suicidal watch. He survived the ordeal with no significant neurological sequela and was discharged from the hospital after around four weeks of the initial event with excellent follow-up in the outpatient department. He refused to partake in a psychiatric evaluation and reported better overall mental health after his family came to join him.

## Discussion

Among people who commit suicide, drug combinations have been historically more effective in achieving the intention [6]. A previous study found a clinically significant increase in the rate of organ support interventions (vasopressors, mechanical ventilation, ICU admission, etc.) when co-ingestions are present. In this case, the patient was denied candidacy for ECMO support for severe vasoplegic shock due to a comatose state with no brainstem reflexes. This state was mediated by the co-ingestion of baclofen, which was realized upon the resolution of the toxicity and return of brainstem functions. Such cases have been previously documented in the literature, but in our search, we found no previously documented cases in which baclofen was the co-ingested substance [7].

While substantial literature exists to support the management of calcium channel blocker toxicity, baclofen represents a challenge to manage, with no large pool of cases from which management strategies can be derived. The most important challenge remains the identification of the toxicity, especially in unwitnessed ingestions, as in the case of suicidal intentions or in the elderly with polypharmacy. In our case, the presentation of an obtunded state accompanied by severe vasoplegic shock led the team to stray from the possibility of co-ingestion and focus on hemodynamic management, as well as the expectation of severe neurologic damage due to hypoperfusion/hypotension. When the most feasible test, EEG, was obtained, it showed the presence of electric activity. This goes against brain death, which caused a dilemma to the treating team [8]. ECMO was considered during the initial resuscitation phase but was withheld due to the severe expected neurologic dysfunction. Upon the obtainment of the EEG, it was realized that the presentation may be more insidious than expected, and neurology services shared their concerns regarding the possibility of a secondary insult that is causing this depression in the level of consciousness. After the effects of baclofen resolved from the system, the patient started to regain consciousness and was interrogated regarding the ingestion that led to the hospitalization, upon which he admitted to a co-ingestion of around 40 tablets of baclofen in addition to the calcium channel blocker.

Baclofen is a known agonist of gamma-aminobutyric acid (GABA) B receptors on presynaptic and postsynaptic neurons in the central nervous system [9]. It was initially developed to treat seizures with poor performance; however, it showed good results in treating muscle spasticity and was approved by the Food and Drug Administration (FDA) for this indication through multiple routes [10]. It has been in use for a long time for multiple off-label uses, which include alcohol use disorder, gastroesophageal reflux disease, and narcolepsy, among others [11-13]. While baclofen use as an oral agent has been known to be relatively safer than the historically more frequent intrathecal administration, toxicity remains a concern, especially with self-harm intentions. The mechanism behind the toxicity of baclofen remains unclear and poorly understood, but the major theories emphasize the overactivation of the GABAergic and glutamatergic systems. This is thought to be a result of neural excitation of the presynaptic and postsynaptic inhibitory interneurons due to hyperpolarization, which leads to a lowered seizure threshold [14]. In addition, many case reports describe the complete loss of brainstem reflexes, both burst suppression and/or non-convulsive status epilepticus, in addition to moderate-to-severe generalized slowing. All these findings can impair the clinical reasoning process as well as conflict with the diagnostic process/management. The best approach remains to deal with these presentations as two separate events unrelated to each other [15-18]. This ingestion had also been further complicated by severe vasoplegic shock due to toxic ingestion of calcium channel blockers, causing severe hemodynamic instability.

Calcium channel blockers have been extensively discussed and reviewed in prior literature with established and well-studied guidelines encompassing the entire spectrum of mild toxicity requiring observation all the way to multiorgan failure and extracorporeal membrane support [19]. Although veno-arterial extracorporeal membrane oxygenators (VA-ECMO) have been a standard rescue therapy for patients presenting with terminal vasoplegic shock, as our patient, the use of such intervention was evoked from possible interventions due to the expected neurological demise evident by the neurological examination. Invasive and life-sustaining interventions such as VA-ECMO present a unique and difficult challenge, particularly regarding the time of deployment. There is a lack of literature to answer this difficult question, but one meta-analysis with good outcomes showed that the average median time to ECMO was ~17 hours, with a survival to discharge of around 60% [20]. We strongly believe a multidisciplinary team approach involving an ECMO-trained physician is the most reasonable approach to determine candidacy and ideal cannulation time.

Our case report is not without limitations, such as our inability to quantify the extent of the ingestion and the unavailability of serum titers for our suspected toxins, but this sheds light on the importance of clinical intuition. We do believe the clinician must rely solely on solid data and ancillary testing, but the experienced physician’s gestalt remains an invaluable tool to probe the presentation thoroughly and ensure all the pieces of the story fall in place. Our patient presented with suspected massive ingestion of a calcium channel blocker and was neurologically unstable to the point of suspected brain death, but careful clinical assessment proved otherwise, and even though invasive mechanical circulatory support measures were withheld, he made a full recovery and was discharged from the hospital in excellent condition. Future research should focus on ways to identify and quantify the possibility of baclofen toxicity, such as serum measurements or clinical scores to elicit suspicion, not just for baclofen but for combined toxicities.

## Conclusions

Toxicological presentations pose unique and challenging aspects that may burden the general intensivist. Patients are often young, with underlying psychiatric conditions that may complicate the average ingestion. Ingestions can be single-drug ingestions or combined-drug ingestions, which further escalates the complexity. The initial encounter can be very dramatic and emotional, and history is often unreliable, especially in unwitnessed ingestions. A complete and thorough toxicologic examination in conjunction with a trained toxicologist can aid in dissecting such a complex presentation and should be sought immediately when available. As the event we encountered has not been reported to date, identifying the nature of it is difficult in our setting; however, it is our consensus that it was two separate toxicologic presentations in one patient due to a single-time combined ingestion rather than a synergistic effect of both medications together, which we reached after a department-wide discussion of the case.
